# Renal artery thrombosis in Behçet’s disease: Case report and literature review

**DOI:** 10.1097/MD.0000000000043740

**Published:** 2025-08-01

**Authors:** Sally Abi Dargham, Oussama Fakih, Sara Hachem, Ouidade Aitisha Tabesh, Joe El-Khoury, Fouad Fayad

**Affiliations:** a Department of Rheumatology, Lebanese Hospital Geitaoui-UMC, Faculty of Medical Science-Lebanese University, Beirut, Lebanon; b Department of Internal Medicine, Division of Rheumatology, Lebanese American University Gilbert and Rose-Marie Chagoury School of Medicine, Byblos, Lebanon; c Department of Radiology, Lebanese Hospital Geitaoui-UMC, Beirut, Lebanon.

**Keywords:** acute kidney injury, angioplasty, case report, renal artery thrombosis and stenosis, thrombectomy, vascular Behçet

## Abstract

**Rationale:**

Behçet disease (BD) is a multisystem inflammatory vasculitis, which can affect all types and sizes of blood vessels, commonly superficial vein thrombosis, deep vein thrombosis, and pulmonary aneurysm. However, renal artery thrombosis has not been yet reported. This case highlights a rare vascular complication of BD.

**Patient concerns:**

A 47-year-old male patient diagnosed with BD, hypertension, dyslipidemia, coronary artery disease, erectile dysfunction, and gout, presented to the emergency department for severe continuous left flank pain unrelieved by positional change, which started 3 days ago and rapidly increased in intensity. Imaging and laboratory studies revealed the presence of left renal artery thrombosis.

**Diagnoses:**

Left main renal artery thrombosis due to Behçet vasculitis.

**Interventions:**

Endovascular thrombectomy, anticoagulation, and immunosuppressive drugs. A relapse of left renal artery stenosis occurred 6 months later treated by angioplasty and stenting.

**Outcomes:**

The patient is currently under follow-up observation.

**Lessons:**

A high index of suspicion of renal artery thrombosis should be kept in mind in cases of patients with BD presenting with unilateral flank pain.

## 1. Introduction

Behçet disease (BD) is a systemic auto-inflammatory vasculitis characterized by a chronic relapsing and remitting course. The highest prevalence is observed along the historical Silk Road, spanning from the Mediterranean Basin to East Asia, which is attributed to the distribution of the HLA-B51 gene.^[1]^ This association has earned BD the historical name “Silk Road disease.”

The etiology of BD involves a complex interplay between environmental and genetic factors, particularly epigenetic alterations that disrupt T-cell homeostasis and trigger chronic inflammation.^[2,3]^ Clinically, BD presents with a heterogeneous spectrum of manifestations, including mucocutaneous, ocular, articular, vascular, neurological, and gastrointestinal involvement.^[4]^ Diagnosis relies entirely on clinical presentation and phenotypic assessment, as no specific laboratory, radiological, or histological markers are available to confirm the disease.^[5]^ Recurrent oral aphthous ulcers are the hallmark feature of BD and are present in 95% of cases, often preceding other symptoms.^[6]^

Vascular involvement in BD occurs in 5% to 40% of cases and is more prevalent in males.^[7]^ It is a significant predictor of morbidity and mortality.^[8]^ Venous complications are more common than arterial ones, with thrombophlebitis being the most frequent venous manifestation and aneurysms being the predominant arterial complication.^[9]^ Renal artery involvement in BD is exceptionally rare. While renal artery stenosis has been documented in isolated cases, renal artery thrombosis has not been previously reported in the literature. This case report highlights a unique presentation of renal artery thrombosis superimposed on renal artery stenosis in a young male patient with BD.

## 2. Case presentation

A 47-year-old male with a known history of BD, managed with colchicine 1 mg daily, hypertension controlled with candesartan and hydrochlorothiazide daily, dyslipidemia, coronary artery disease, erectile dysfunction, and gout, presented to the emergency department with severe, continuous left flank pain radiating to the left groin, unrelieved by positional change. The pain had developed gradually over 3 days, peaking a few hours prior to presentation. He reported no history of recent trauma or fever. Three weeks prior, he had a mild COVID-19 infection, confirmed by a rapid antigen test, which resolved with symptomatic treatment and did not require corticosteroids. His medical history included recurrent renal stones treated twice with the placement of a double-J catheter 2 years prior. He also had an episode of superficial vein thrombosis (SVT) in the right ankle 4 years prior, followed a few months later by a deep vein thrombosis (DVT) in his right calf, which was successfully treated with anticoagulants. In addition, the patient received intramuscular testosterone injections, administered monthly for 3 months, prescribed by a sexologist for erectile dysfunction, with the last dose given 2 weeks prior to presentation.

Upon presentation, vital signs revealed a high blood pressure of 167/107 mm Hg and a heart rate of 99 beats per minute. The respiratory rate was 18 respirations per minute, with an oxygen saturation of 97%. He was afebrile, and physical examination showed guarding upon deep palpation of the left lower abdominal quadrant, along with left costovertebral angle tenderness.

Laboratory tests indicated an elevated white blood cell count of 13,840/mm³ with neutrophilia (83%), an elevated C-reactive protein of 139 mg/L, and an elevated serum creatinine of 1.74 mg/dL (estimated glomerular filtration rate [eGFR] of 48 mL/min). Blood urea nitrogen was normal at 25 mg/dL. Lactate dehydrogenase was slightly elevated at 265 mg/dL (normal < 248), and D-dimer was elevated at 678 ng/mL (normal < 500). Liver function tests were within normal limits, with aspartate aminotransferase at 32 U/L, alanine aminotransferase at 22 U/L, alkaline phosphatase at 45 U/L, and gamma-glutamyl transferase at 49 U/L. Total bilirubin was 0.3 mg/dL, and direct bilirubin was 0.1 mg/dL. Albumin was 4.0 g/dL. Blood coagulation tests were also normal, with a prothrombin time of 11.5 seconds, activated partial thromboplastin time of 25 seconds, and an international normalized ratio of 0.97. Urinalysis was normal, and other laboratory tests were unremarkable.

**Figure 1.**
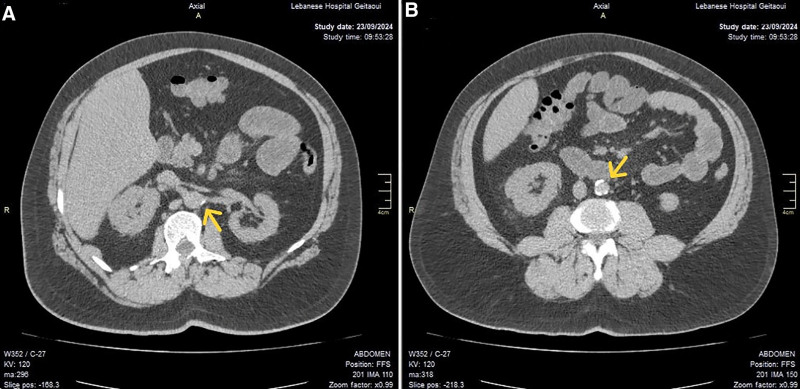
CT of the abdomen and pelvis without IV contrast showing calcifications on the origin of the left main renal artery (A) and the abdominal aorta (B).

A computed tomography (CT) scan of the abdomen and pelvis, performed without intravenous contrast, showed kidneys of normal size without evidence of urinary tract calculus or hydronephrosis. Calcified atherosclerosis was noted in the aorta and left main renal artery (Fig. 1).

**Figure 2.**
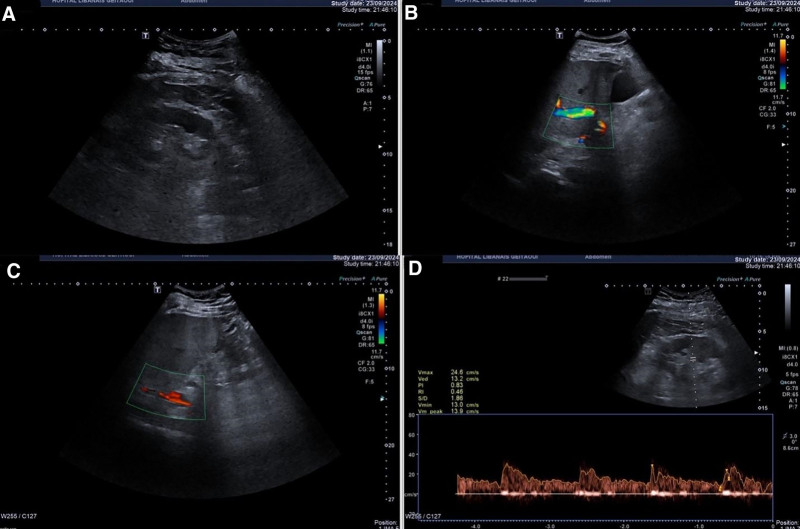
(A–D) Doppler ultrasound of the abdomen and pelvis with arterial and venous spectral waveforms at the level of the kidneys showing no evidence of renal vein thrombosis.

Urgent Doppler ultrasound of the abdomen and pelvis yielded inconclusive results, as arterial and venous spectral waveforms were registered at the level of the kidneys (Fig. 2).

**Figure 3.**
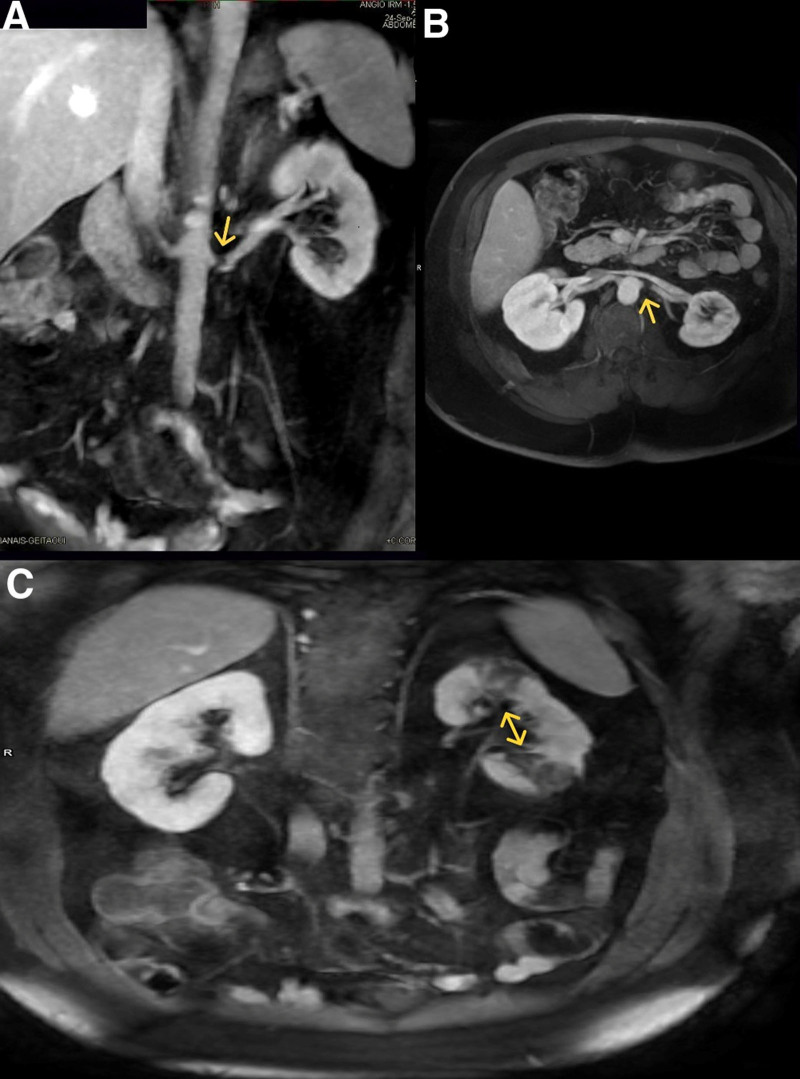
Magnetic resonance angiography of the abdomen and pelvis showing the sub-occlusive thrombus in the left main renal artery (A, B), multiple wedge-shaped renal infarcts (C).

The next day, a magnetic resonance angiography of the abdomen and pelvis was performed and revealed a 7 to 8 mm sub-occlusive thrombus in the left main renal artery, 5 mm distal from its origin. Multiple wedge-shaped parenchymal defects involving both the cortex and the medulla were also observed, consistent with renal infarcts (Fig. 3).

**Figure 4.**
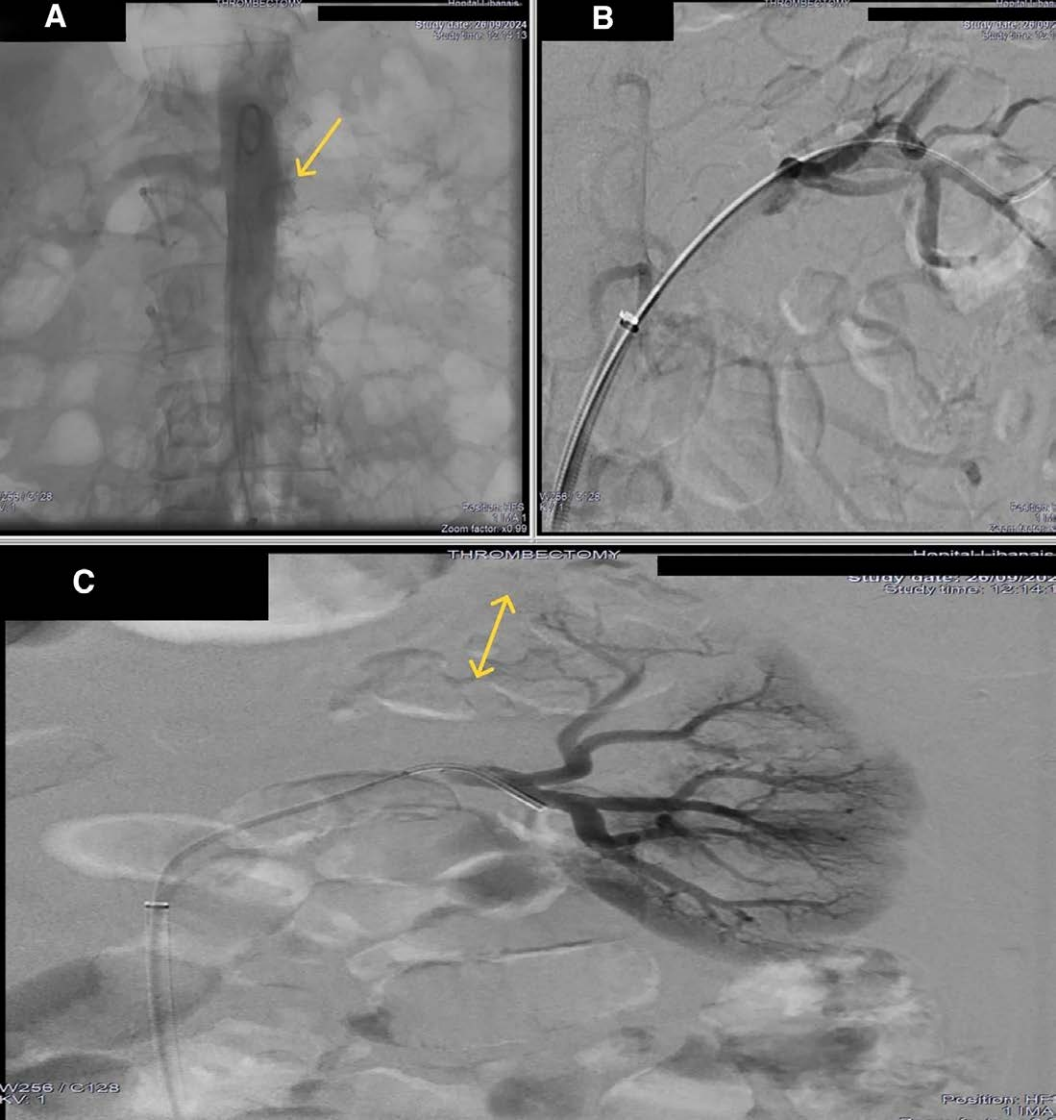
Aortogram showing complete thrombosis of the left renal artery (A), successful catheterization (B) and decreased flow in the renal vasculature (C).

**Figure 5.**
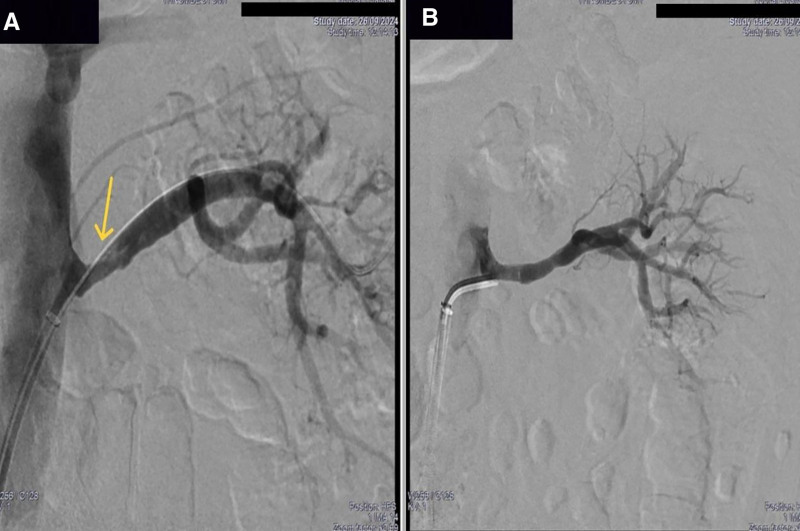
Mechanical thrombectomy showing focal stenosis at the site of the aspirated thrombus (A), post-thrombectomy balloon angioplasty restoring full patency (B).

The patient was started on therapeutic anticoagulation with unfractionated heparin, in addition to intravenous pulse methylprednisolone 1 g daily for 3 days, followed by an oral tapering regimen with prednisone. On the third day, an endovascular thrombectomy of the left main renal artery was planned. However, the initial angiogram showed a complete thrombosis of the left renal artery. The artery was successfully recanalized with subsequent aspiration of a large quantity of fresh and chronic thrombus using an 8F mechanical automated aspiration device (Penumbra®, CA). Post-aspiration control angiogram showed a very severe focal post-ostial artery stenosis, which was treated successfully with an angioplasty using a 6-mm non-compliant balloon (Figs. 4 and 5). Stenting was deemed unnecessary, especially considering the non-atheromatous nature of the stenosis and the patient’s young age.

Thrombophilia work-up, including anti-cardiolipin antibodies, lupus anticoagulant, anti-β2 glycoprotein antibodies, protein C, protein S, and antithrombin III, was negative. Serologic testing for hepatitis B virus, hepatitis C virus, and tuberculosis infection were also negative.

The final diagnosis was acute renal artery thrombosis on top of renal artery stenosis as a complication of the underlying vasculitis secondary to BD. Systematic follow-up on the endovascular thrombectomy and angioplasty of the left main renal artery by Doppler ultrasound 1 week later showed no gross parenchymal ischemic defects, with normal global vascularization, patent renal arteries bilaterally, and a normal intra-renal resistive index ranging from 0.54 to 0.57 (normal < 0.7). Furthermore, the serum creatinine level decreased to 1.31 mg/dL by day 4 after the procedure. The patient’s pain improved significantly, and he was pain-free by day 1 post-procedure, with blood pressure dropping to below 140 mm Hg systolic by day 2.

**Figure 6.**
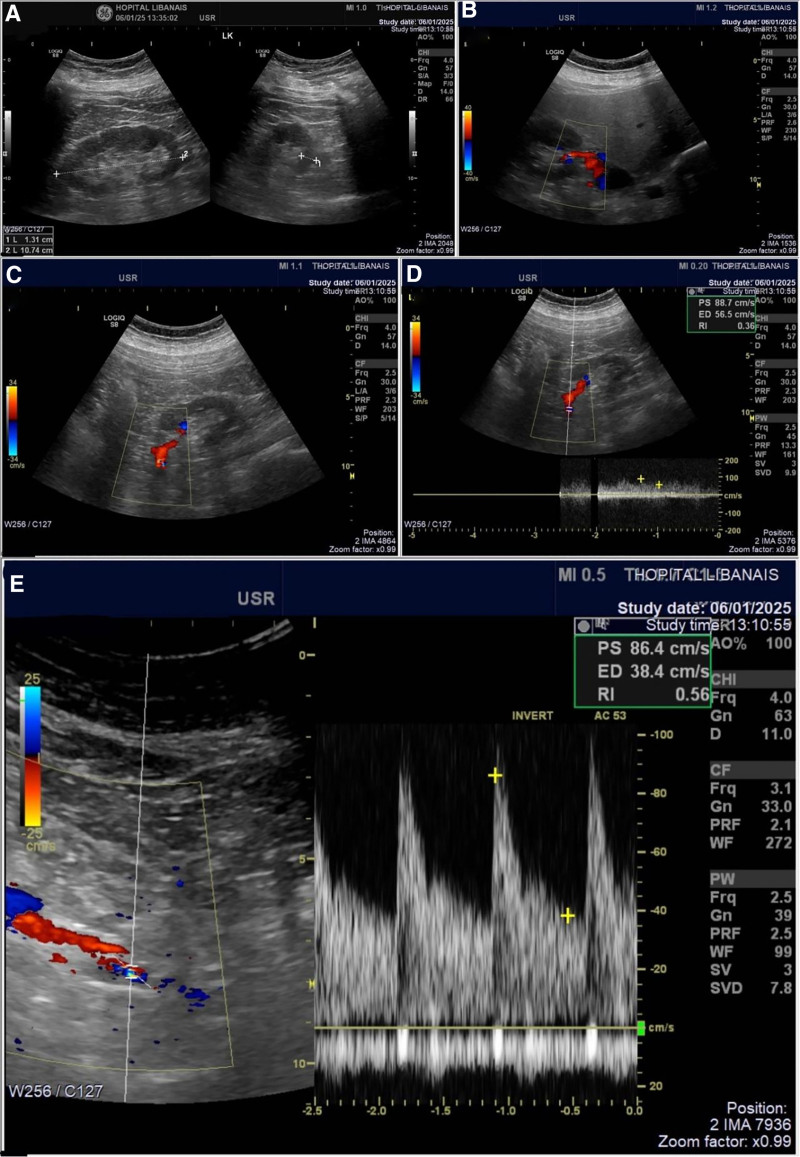
(A–E) Renal Doppler ultrasound showing normal patency of the renal arteries and veins bilaterally with normal global vascularization.

On day 8, the patient received the first IV dose of infliximab (anti-TNFα) 300 mg (3 mg/kg), with subsequent administrations following the infliximab protocol. He was discharged on apixaban 10 mg twice daily, aspirin 100 mg daily, an oral corticosteroid tapering regimen, in addition to his previous home medications. At a 3-month follow-up visit, the patient had a complete resolution of symptoms, a normal renal Doppler ultrasound (Fig. 6), and a return to baseline kidney function, with a serum creatinine of 1.13 mg/dL (eGFR 81 mL/min using CKD-EPI 2021).

**Figure 7.**
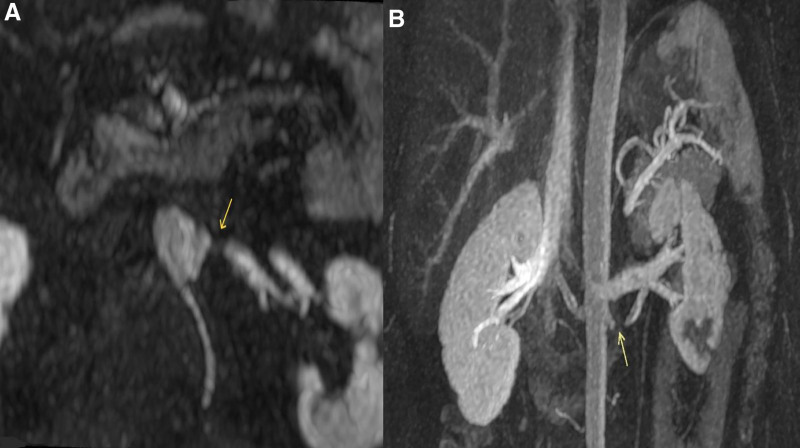
(A and B) Magnetic resonance angiography of the abdomen showing a severe post-ostial stenosis.

**Figure 8.**
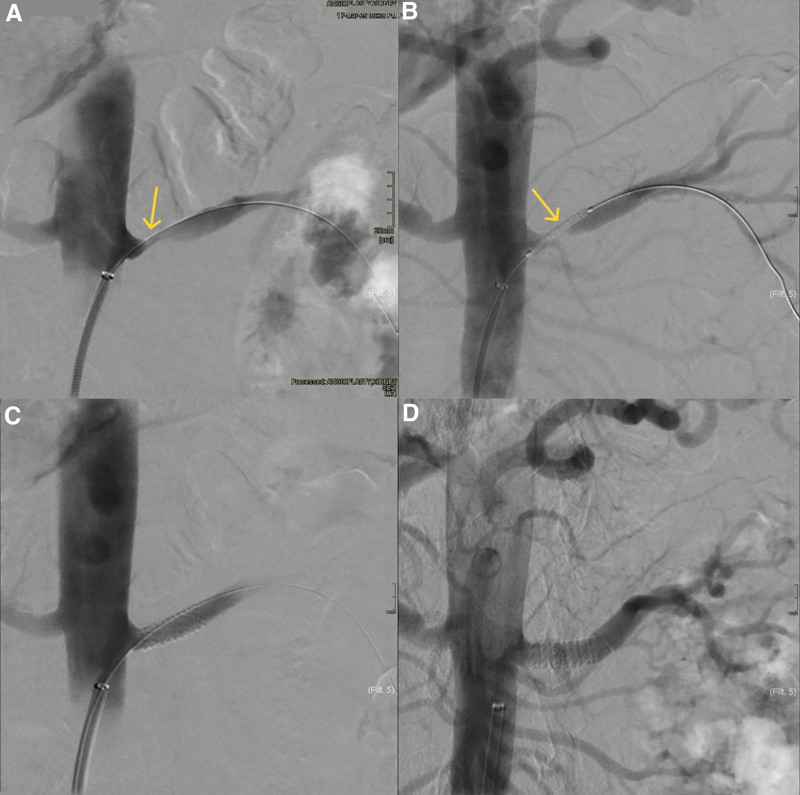
Catheterization of the stenotic renal artery (A) with subsequent balloon dilatation and stent insertion (B, C), control angiogram after stent insertion showing patent flow without residual stenosis (D).

However, at a 6-month follow-up visit, laboratory studies showed an elevated serum creatinine of 1.46 mg/dL (eGFR 59 mL/min using CKD-EPI 2021) without any clinical symptoms. magnetic resonance angiography of the renal arteries (Fig. 7) was performed, revealing a post-ostial restenosis of the left renal artery 5 mm distant from the ostia, decreasing the flow of contrast to the left kidney. Consequently, the patient was admitted to the hospital and started on clopidogrel 75 mg once daily, in addition to aspirin 100 mg daily, and again received a course of intravenous pulse methylprednisolone of 1 g daily for 3 days. The dosage of infliximab per infusion was increased to 500 mg (5 mg/kg). Three days following admission, successful angioplasty and stenting (7-mm stent) of the affected renal artery segment was performed. The final control angiogram showed a very good result without any residual stenosis and without parietal damage (Fig. 8). Serum creatinine level decreased to 1.35 mg/dL (eGFR 65 mL/min using CKD-EPI 2021) upon discharge 1 day following the procedure, and the 2-week lab follow-up showed creatinine stable at 1.37 mg/dL (eGFR 64 mL/min using CKD-EPI 2021) with a CRP of 1.1 mg/L.

Currently, 9 months after the event, the patient remains clinically stable during follow-up visits and teleconsultations, with stable serum creatinine levels. The patient continues to receive infliximab infusions at a dose of 500 mg (5 mg/kg), which have been well tolerated, with no reported side effects.

## 3. Discussion

Vasculo-BD can affect both arteries and veins. Venous involvement is observed in 15% to 40% of cases, considerably more prevalent than arterial disease, which is found in 3% to 5% of BD patients.^[10]^ Venous disease most commonly manifests as thrombophlebitis, including SVT and DVT, while arterial disease typically presents as aneurysms, particularly pulmonary artery aneurysms; the aorta and peripheral arteries are less frequently affected.^[11]^ An analysis of vascular involvement in 460 patients by Torgutalp et al revealed that superficial thrombophlebitis is the strongest risk factor for subsequent major vascular events.^[12]^ Correlating these findings with the presented case, our patient had his first episode of SVT 4 years prior to the renal artery involvement. Arterial involvement, particularly extra-pulmonary, usually occurs at a later stage, 5 to 10 years after disease onset, as shown in a retrospective study by Tascilar K et al.^[13]^ Thus, this patient presented with relatively early arterial involvement in the disease course. The patient is positive for the HLA-B51 gene, which is associated with a more aggressive form of the disease, particularly vascular involvement.^[14]^

Renal involvement in BD is a rare complication. A retrospective analysis by Zheng W et al elucidated renal damage in 2.6% of 618 hospitalized patients over 14 years. Renal manifestations included renal artery stenosis in 8 patients, chronic glomerulonephritis in 6 patients, renal vein thrombosis in 1 patient, and renal tubular acidosis in 1 patient.^[15]^ Notably, renal artery thrombosis has been exceedingly rare, with no prior reports in the literature, making this case unique. Our patient presented with renal artery thrombosis superimposed on renal artery stenosis; the removal of the thrombus via mechanical automated thrombectomy revealed a substantial quantity of both fresh and chronic thrombi. Toprak O et al reported the first case of renal artery stenosis in a patient with BD successfully treated with angioplasty and stent implantation in 2007.^[16]^ Additionally, Kingushi S et al reported a case of BD presenting as renovascular hypertension due to renal artery stenosis.^[17]^ Upon presentation, our patient also exhibited high blood pressure, likely attributable to the underlying renal artery subtotal obstruction. However, hypertension was not the primary mode of revelation; his blood pressure returned to baseline after the procedure and resumption of antihypertensive therapy.

The initial clinical picture suggested a preliminary diagnosis of renal colic, particularly given the patient’s history of recurrent renal stones and double-J catheter placement. Consequently, an urgent CT scan of the abdomen and pelvis without contrast was performed, ruling out renal calculi. Poggiali E et al reported a case of renal artery thrombosis mimicking renal colic,^[18]^ emphasizing the importance of considering renal artery thrombosis and infarction in the differential diagnosis for patients presenting with a triad of high risk for thromboembolic events, persistent flank, abdominal, or lower back pain, and elevated levels of serum lactate dehydrogenase and/or hematuria.^[19]^ Our patient met all criteria of this triad, being at higher risk for thromboembolic events due to known vasculitis, presenting with persistent left flank pain, and exhibiting elevated lactate dehydrogenase levels. Other rare vascular manifestations of BD include coronary artery pseudoaneurysm,^[20]^ right common iliac artery stenosis,^[21]^ renal artery pseudoaneurysm,^[22]^ and renal arteriovenous fistula.^[23]^

The correlation between thrombotic events and coronavirus disease (COVID-19) has been well established. A study by Idilman IS et al evaluating lung and kidney perfusion abnormalities in patients with COVID-19 using dual-energy computed tomography demonstrated perfusion deficits in 50% of kidneys assessed and 25.8% of lungs assessed.^[24]^ These manifestations are attributable to systemic microangiopathies and microthrombi induced by the hypercoagulable state resulting from COVID-19 infection.^[25]^ Approximately 35 cases of renal artery thrombosis have been reported in the literature, diagnosed within a month of SARS Cov-2 infection.^[26]^ Given that our patient had a COVID-19 infection 3 weeks prior to presentation, albeit resolved without treatment, it may have further increased his risk for thromboembolic events. Moreover, the association between testosterone replacement therapy and thromboembolic events in healthy individuals remains controversial. A retrospective cohort study by Sharma et al found no statistically significant difference in the incidence of DVT/pulmonary embolism between individuals receiving testosterone replacement therapy and those who did not.^[27]^ However, an increased incidence of venous thromboembolic events has been observed among patients with a thrombophilia-hypofibrinolysis profile, with the risk peaking 3 months after initiating testosterone injections.^[28]^ In our patient, thrombophilia work-up was negative. Therefore, it is less likely that testosterone injections significantly increased his risk for a thromboembolic event, though the possibility cannot be entirely excluded.

Due to the rarity of renal artery thrombosis, well-established treatment guidelines are lacking. Management focuses on rapidly restoring kidney perfusion using systemic anticoagulation, percutaneous interventional therapy, or surgery. Successful renal artery thrombectomy and kidney reperfusion via thrombolysis, with a return to baseline kidney function, have been reported.^[29,30]^ Yousif et al and Li et al described successful treatment of unilateral renal artery embolism using mechanical aspiration thrombectomy,^[31,32]^ which was the approach employed in our case, alongside apixaban, a direct oral anticoagulant. While Parekh et al suggest that human kidneys can tolerate only 30 to 60 minutes of ischemia,^[33]^ case reports by Li et al and Gao et al have documented recovery of kidney function after prolonged ischemic periods of up to twenty days and 1 week, respectively.^[32,34]^ In our case, mechanical thrombectomy was performed 3 days after symptom onset and yielded a satisfactory immediate result. The subtotal nature of the thrombotic occlusion and the initiation of unfractionated heparin 2 days prior to the procedure likely contributed to the positive outcome. Furthermore, the patient underwent left renal artery angioplasty with stenting 6 months after the initial procedure due to restenosis.

The 2018 update of the European Alliance Of Associations For Rheumatology recommendations for the management of BD advised the administration of anti-TNF monoclonal antibodies in cases of arterial involvement with a refractory course.^[35]^ A comparative study by Saadoun D et al demonstrated that infliximab had a higher response rate and fewer adverse events compared to cyclophosphamide for induction therapy in severe BD.^[36]^ Moreover, Hatemi et al showed the efficacy of infliximab, with a remission rate of 73% at month 6 and 63% at month 12 in 127 patients with vascular Behçet refractory to immunosuppressants, immunomodulators, and glucocorticoids.^[37]^ Consequently, infliximab was administered to our patient. During the second admission, a more aggressive treatment approach with a higher dose of infliximab was employed. The efficacy of infliximab dose escalation has been demonstrated in various auto-inflammatory and autoimmune diseases, including ulcerative colitis and Crohn disease.^[38]^ Furthermore, a randomized controlled trial by Syversen et al demonstrated the efficacy of therapeutic drug monitoring in sustaining disease control over 52 weeks in immune-mediated inflammatory diseases.^[39]^

## 4. Conclusion

Renal artery thrombosis is a rare condition with a presentation that can mimic various pathologies, necessitating a high index of suspicion, particularly in patients with vasculitis such as BD, even though vascular involvement in BD more commonly manifests as DVT, superficial vein thrombosis, and arterial aneurysms. Diagnosis hinges on meticulous integration of imaging and laboratory findings. Prompt management is crucial to minimize the extent of kidney damage and preserve renal function. This case underscores the importance of considering renal artery thrombosis in the differential diagnosis of flank pain in patients with BD advocating for timely intervention to improve patient outcomes.

## Author contributions

**Conceptualization:** Sally Abi Dargham, Oussama Fakih, Sara Hachem, Fouad Fayad.

**Methodology:** Ouidade Aitisha Tabesh, Fouad Fayad.

**Project administration:** Fouad Fayad.

**Resources:** Sally Abi Dargham, Oussama Fakih, Sara Hachem, Joe El-Khoury.

**Supervision:** Fouad Fayad.

**Validation:** Ouidade Aitisha Tabesh, Joe El-Khoury, Fouad Fayad.

**Visualization:** Joe El-Khoury.

**Writing – original draft:** Sally Abi Dargham, Oussama Fakih, Sara Hachem, Ouidade Aitisha Tabesh, Joe El-Khoury, Fouad Fayad.

**Writing – review & editing:** Sally Abi Dargham, Oussama Fakih, Sara Hachem, Ouidade Aitisha Tabesh, Joe El-Khoury, Fouad Fayad.
